# 

**Published:** 1885-11

**Authors:** 


### CONTENTS

ORIGINAL COMMUNICATIONS—

Syphilis, the Importance of a Thorouh Diagnosis and Long-Continued Treatment. By J. C. LeHardy, M. D., Savannah, Ga... 513
Experimental-Duodeno Cholecystostomy. By J. McF. Gaston, M. D., Atlanta, Ga .......... 533

SOCIETY REPORTS—

Macon Medical Society .......... 539

CLINIC REPORTS—

Epithelioma and Varicose Ulcer. By Henry Wile, M. D.......... 544

CORRESPONDENCE—

Letter from Augusta. The Local Option Act and the Medical Profession. By Wm. H. Doughty, M. D., Augusta, Ga.......... 550
Our Philadelphia Letter. By Harry Huzza.......... 555

BOOK REVIEWS—

Handbook of General Therapeutics. By H. Von Ziemssen, M. D. 560
Practical Treatise on Nasal Catarrh. By Beverly Robinson, M. D. 562
The use of the Microscope. By Dr. Carl Friedlander.......... 563
Inebriism, a Pathological and Psychological Study. By T. L. Wright, M. D.......... 563
Insomnia, and other Disorders of Sleep. By H. M. Lyman, M. D. 564
Epitome of Diseases of the Skin. By Louis A. Duhring, M. D... 564
A Treatise on Nervous Diseases. By S. G. Webber, M. D.......... 565
The Management of Labor and the Lying-in Period. By H. G. Landis, M. D.......... 565
A Practical Treatise on Diseases of Children. By Alfred Vogel, M. D.......... 566
Books and Pamphlets Received.......... 566

EDITORIAL—

The Local Option Act.......... 569
Items.......... 549, 572

